# Simulation of encounter rates between zooplankton organisms and microplastics in a tropical estuary

**DOI:** 10.1371/journal.pone.0292462

**Published:** 2023-10-05

**Authors:** Laura Sanvicente-Añorve, Miguel Alatorre-Mendieta, Mitzi Sánchez-Campos, Guadalupe Ponce-Vélez, Elia Lemus-Santana

**Affiliations:** 1 Laboratorio de Ecología de Sistemas Pelágicos, Instituto de Ciencias del Mar y Limnología, Universidad Nacional Autónoma de México, Mexico City, Mexico; 2 Laboratorio de Oceanografía Física, Instituto de Ciencias del Mar y Limnología, Universidad Nacional Autónoma de México, Mexico City, Mexico; 3 Posgrado en Ciencias del Mar y Limnología, Universidad Nacional Autónoma de México, Mexico City, Mexico; 4 Laboratorio de Contaminación Marina, Instituto de Ciencias del Mar y Limnología, Universidad Nacional Autónoma de México, Mexico City, Mexico; Central University of South Bihar, INDIA

## Abstract

Coastal estuarine systems may hold a large number of microplastic particles, which predators often mistake for prey. This study estimated the encounter rates between microplastics (alleged prey) and zooplankton having different feeding modes, trophic positions, swimming velocities, and perception distances, under calm and turbulent conditions, and during two seasons. Surface water samples were taken at 10/12 sites of the Sontecomapan lagoon, southern Gulf of Mexico, to quantify microplastic concentration. Zooplankton organisms considered were copepods, chaetognaths, and luciferids, common organisms in the lagoon. In June, at surface waters and during calm conditions, mean encounter rates were 1.5, 2450, and 980 particles per individual per hour, that is, for copepods, chaetognaths, and luciferids, respectively. When the wind blows (0.8 m s^-1^) encounter rates were 1.2, 1.4, and 2.6 times higher than in calm conditions. In October, mean encounter rates under calm conditions were 0.2, 355, and 142 particles per individual per hour, for copepods, chaetognaths, and luciferids; these values increase 1.3, 1.6, and 3.3 times when the wind blows (1.12 m s^-1^). The major number of encounters in June was due to a higher concentration of microplastics, despite the lower turbulent velocity. Regarding their trophic position, we propose that secondary consumers (chaetognaths and luciferids) are more affected because they could eat microplastics via contaminated prey or accidentally ingest them owing to confusion in the motion signals, especially under turbulent conditions. Another consequence of encounters could be the entanglement of microplastics in the body of the animals, especially in those with complex morphology, such as crustaceans. Encounters between zooplankton and microplastics do not always result in ingestion or entanglement, but the encounters are the first step in the case of occurrence.

## Introduction

Estuaries provide valuable ecosystem goods and services for people, such as food production, shipping routes, recreational activities, storm protection, and the production of chemicals used in pharmacy, among others [1, 2]. Despite the multiple and significant benefits provided to humans, estuaries are viewed as among the most threatened ecosystems in the world due to anthropogenic impact [3]. Human contaminant activities around estuaries, or near the tributaries feeding them, even located hundreds of kilometers away, may damage these ecosystems [4]. Terrestrial inputs may contain a wide variety of pollutants, from which microplastics are considered emerging threat contaminants [5, 6].

Microplastic debris is found in all aquatic environments around the world and represents a considerable risk to the health of marine and freshwater ecosystems and their associated biota [7, 8]. Microplastics enclose any synthetic solid particle of a wide range of polymers of irregular or regular shape and between 1 μm to 5 mm in size [9, 10]. Once released into the basins, microplastics may remain in the water column or be deposited on the bottom, depending on their density. The bioavailability (proportion of the total number of particles in the environment that are potentially available for intake by an organism) of microplastics to pelagic biota mainly depends on their size, shape, density, abundance, and color, as well as the feeding modes of organisms [7, 11]. Regarding their size, microplastics could be potentially ingested by a variety of organisms, and the risks they pose to the biota are influenced by the encounter rates between microplastics and organisms [8, 11]. Because of the ubiquity and persistence of microplastics in the aquatic environment, the inhabitants are highly exposed to them. In the water column, this is the case of the zooplankton, which display several feeding modes [12]. The ingestion of plastics involves all trophic levels, from filter feeders, invertebrate grazers, and predators; plastics are also ingested by planktivorous and herbivorous fish, suggesting a trophic transfer and a wider distribution of this contaminant across aquatic food webs [13]. The effects of microplastics on zooplankton have been mostly studied in marine organisms; however, it is expected that species with similar autecology are similarly affected [14]. The potential impact of microplastics on crustacean zooplankton includes a decrease in feeding rates, fecundity, survival, and population growth [14–16]. Additionally, microplastics can be entangled among the external appendages of small crustaceans, limiting the function and behavior of animals, such as motion, feeding, mechanoreceptors, and then, the ability to search for mates, prey, or evade predators [17].

Encounter rates denote the expected number of encounters between a searcher and the targets over a specific geometry and per unit of time [18]. In the water column, encounters between a zooplankter predator and their prey (or alleged prey) are influenced by the swimming velocity of both predator and prey, the abundance of prey, the encounter radius of the predator, as well as the turbulence intensity of the water [19, 20]. Small-scale turbulence interacts in several ways with plankton communities. It affects the plankton distribution [21], nutrient uptake and phytoplankton growth [22], the zooplankton swimming behavior [23], and the rate of plankton sedimentation [24]. Furthermore, turbulence can affect the different stages of the predatory cycle (i.e. search, encounter, detection, attack, and capture; [25]); it enhances encounter rates between predators and prey, but it also decreases the period of contact between them [22]. As passive buoyant particles in the water, microplastics can be mistaken for prey [8], but the interaction is scarcely known. Laboratory experiments have established that zooplankton can ingest small plastic particles [26–28], and recent work has shown this also occurs in wild populations [29–31]; in any case, to ingest a particle, the first step is to encounter it. Taking a tropical Mexican estuary from the southern Gulf of Mexico as the target studied area, in this study we 1) estimated the encounter rates of microplastics with three kinds of zooplankton organisms having different feeding modes, swimming velocities, and perception distances, and 2) analyzed the effect of the turbulence induced by the wind on the encounter rates during two seasons. The results were discussed regarding the food-gathering methods and the trophic level of organisms. This study warns about the threats of microplastics in the estuary and provides a basis for future research on the interaction between microplastics and zooplankton.

## Materials and methods

### Study area

**Fig 1:**
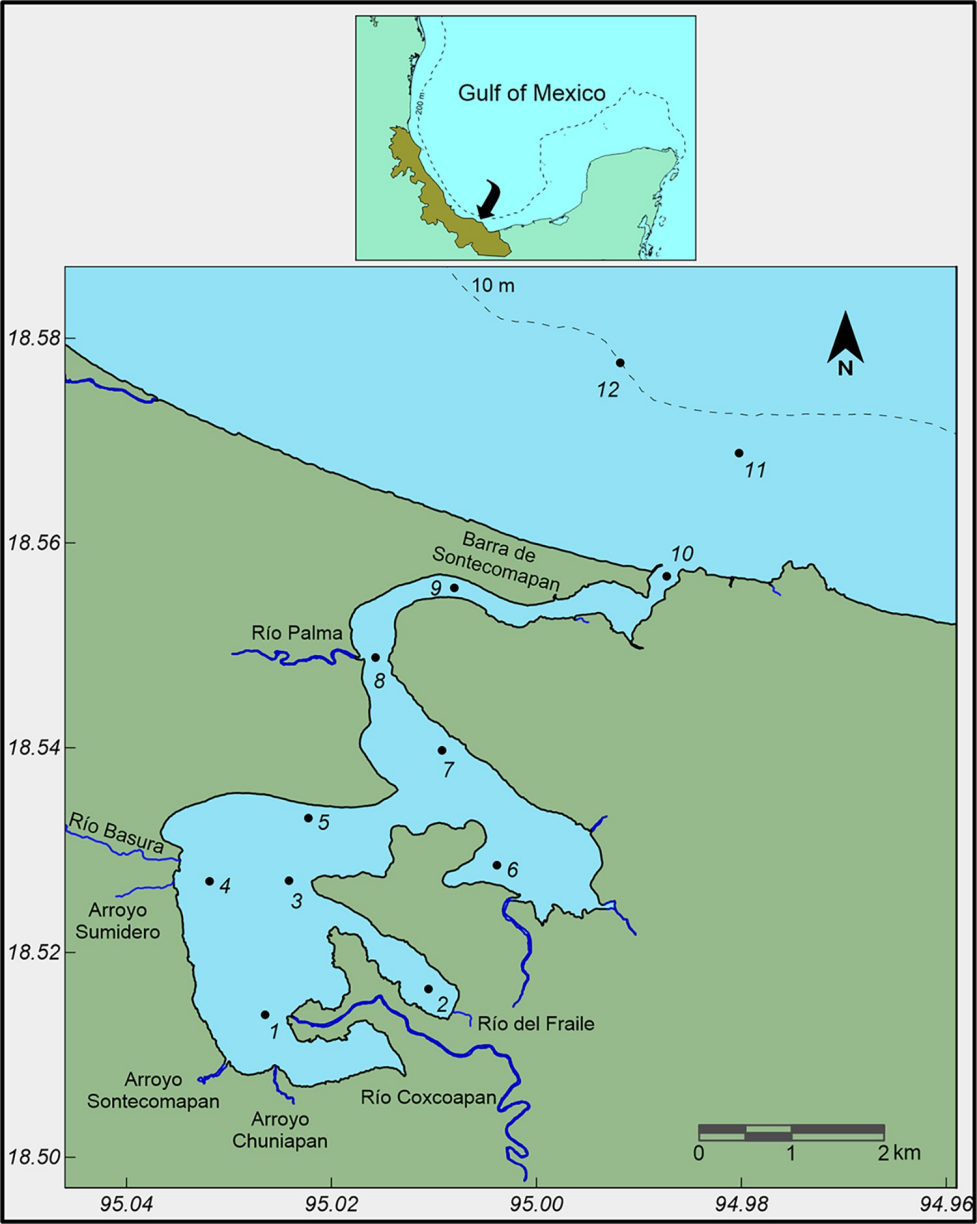
Geographical location of the Sontecomapan lagoon and position of sampling stations.

The Sontecomapan lagoon is located in the Mexican State of Veracruz, between 18.51–18.57° N and 94.99–95.04° W, on the southern margin of the Gulf of Mexico. The lagoon is a natural protected area enlisted in the Ramsar site’s index (site number 1342) and forms part of the Los Tuxtlas Biosphere Reserve. Sontecomapan lagoon has an irregular shape of about 7 km in length, 5 km in width, and 1.5 m in depth (Fig 1). It is permanently connected with the sea by a narrow mouth located in the northeastern portion and several minor rivers, such as Coxcoapan, Palma, and Basura, flow into the lagoon. Here, marine and freshwater mixing leads to variable salinity conditions depending on the volume of freshwater inflow and tides [32]. Sontecomapan is bordered by the mangroves *Rhizophora mangle*, *Laguncularia racemosa*, and *Avicennia germinans* [32] which provide vital microhabitats for the spawning, protection, and development of marine and estuarine fishes. Surrounding vegetation also provides important resting places for resident and migratory birds coming from North America [33]. Tourism and local fishing in the lagoon are important economic activities for the coastal human population. Main fisheries include oysters, white shrimps, prawns, snappers, seabasses, croakers, and mullets [34].

### Sampling and laboratory procedures

Sampling was done in June 2018 and October 2019 in 10/12 sites of the lagoon (Fig 1). To quantify microplastic concentrations in the estuary, surface water was obtained with a 10-liter container, the content was stirred manually, and then, one liter of water was transferred to a 1-liter glass flask and kept at 4°C. In the laboratory, the water was sieved through two coupled sieves (4.75 and 0.053 mm), and the retained material between the sieves was subjected to organic matter oxidation with hydrogen peroxide (30% H_2_O_2_). Afterward, a saturated solution of zinc chloride (933 g L^-1^) was added to carry out separation by density, so that the plastic particles floated. After 4 h the supernatant was passed through a vacuum filtration system (Whatman glass microfiber paper GF/A, 47 mm diameter, 1.6 μm pore size). Each filter was placed in a glass Petri dish and dried in an oven at 40°C for one week [35, 36]. The plastic particles retained in the filter were quantified under a stereoscopic microscope. Microplastic concentrations were expressed as the number of particles per liter of water.

Detection of microplastics was made using a Raman DXR Microscope–Thermo Fisher, under the following conditions: 780 nm infrared laser with a power of 24 mW and a 10X objective with a time exposure of 5 s. An open-source library was consulted to compare collected spectra to reference spectra (https://openanalysis.org/openspecy/).

To avoid external microplastic contamination during laboratory procedures, the work area was cleaned and covered with aluminum foil before the analyses. Also, cotton lab coats and clothing were used, as well as polymer-free gloves. A blank was added for every batch of eight analyzed samples.

### Data analysis

To simulate the encounters with microplastics, we choose three kinds of organisms (copepods, luciferids, and chaetognaths) having different feeding modes and trophic positions. In Sontecomapan lagoon, copepods are mostly represented by *Acartia tonsa*, the luciferids by *Belzebub faxoni*, and the chaetognaths by *Parasagitta friderici*; all of them are common organisms and have a wide distribution in the estuary [37].

The simulation of encounter rates between zooplankton and microplastics was made under two scenarios: non-turbulent and turbulent conditions induced by the wind. Two ecological models were used in the estimation of the encounter rates: the Gerritsen and Strickler model (GS) [19] for the analysis of calm conditions, and the Rothschild and Osborn model (RO) [20] to simulate the small-scale turbulent scenario. Originally, these models were developed to estimate the encounter rates between the predators and prey in the pelagic environment; in this study, the models were used to give an estimation of the encounter rates between some zooplankton organisms (predators) and the microplastics (alleged prey).

The encounter rate in the GS model (*C*_*GS*_) is defined by

$$C_{GS}=\frac{\pi R^{2}N}{6}\frac{{\left(x+y\right)}^{3}-{\left|x-y\right|}^{3}}{xy}$$

where *R* is the encounter radius (m) of predators, *N is* the number of “prey” per m^3^, *x* the “prey” velocity (m s^-1^), and *y* the predator velocity (m s^-1^). The RO model modified the GS model by introducing the small-scale turbulent velocity (*w*) induced by the wind when it blows at surface waters. In this model, the *x* is replaced by $\sqrt{x^{2}+w^{2}}$ and the *y* is replaced by $\sqrt{y^{2}+w^{2}}$. The term *w* can be calculated as the root-mean-square of the turbulent kinetic energy (*k*). The dissipation rate of energy (*ε*, m^2^ s^-3^) was also estimated in the water column. The calculations of the terms *k*, *w*, and *ε* are exposed in previous works [38, 39].

Wind conditions were taken from the Windy Weather Service platform (https://www.windy.com) for the Barra de Sontecomapan station. For June and October, mean wind values were 0.80 and 1.12 m s^-1^, respectively. These values were used to estimate the turbulent velocity (*w*).

For copepods, the swimming speed (*y*) was 6.2 mm s^-1^ and the encounter radius *R*, 0.4 mm [40, 41]; for chaetognaths, the *y* was 4 cm s^-1^, and the *R*, 20 mm [42, 43]. For luciferids, the *y* was 1.6 cm s^-1^ [43] and the *R* was assumed to be 20 mm.

The velocity *x* for microplastics (alleged prey) was taken to be zero. The number of “prey” (or microplastics, *N*) was randomly taken from the set of sampling stations in each season. This bootstrap procedure consisted of drawing a random sample, repeatedly and with replacement, from the observed data set to estimate the parameter. After 1000 repetitions, the mean rate and the standard deviation were calculated. The encounter rates were expressed as the number of microplastic particles encountered by an individual in an hour (example: part copepod^-1^ h^-1^). Our estimations assume no variability in the concentration of microplastics with depth.

## Results

In June, microplastic particles were found in all the ten sampling stations examined, in a range of 7 to 26 part L^-1^ (mean concentration 13.5 ± 7.1 part L^-1^). In October we took twelve samples, but unfortunately, five were broken during the transport; in this month, concentration varied between 0 and 6 part L^-1^ (mean concentration 2 ± 1.7 part L^-1^). The presence of microplastics in the Sontecomapan lagoon was confirmed by spectroscopy.

**Fig 2:**
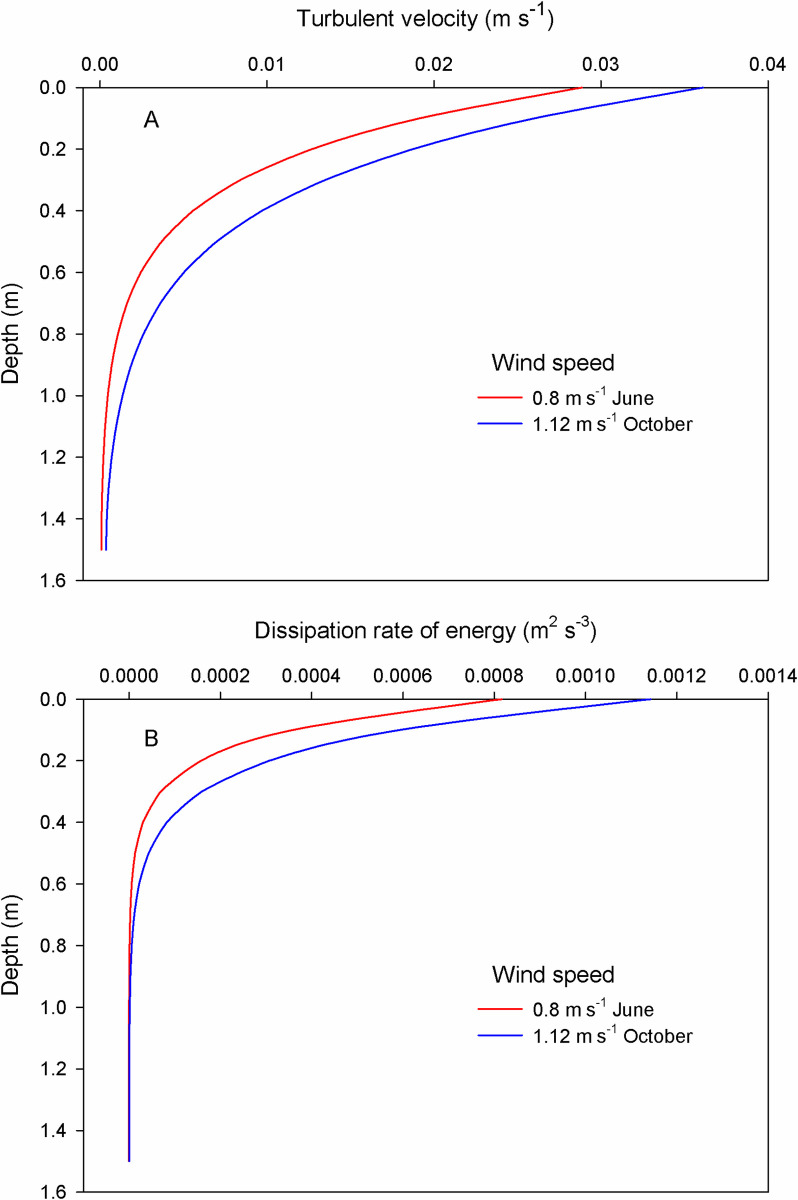
Vertical profiles of turbulent velocity (A) and dissipation rate of energy (B) in the Sontecomapan lagoon considering two wind conditions.

Turbulent velocity (*w*), in June, was 0.03 m s^-1^ at surface waters at a wind speed of 0.8 m s^-1^. In October, this parameter was 0.04 m s^-1^ at a wind speed of 1.12 m s^-1^. Turbulent velocity exponentially decreased with depth until almost zero near the bottom (Fig 2A). The dissipation rate of energy (*ε*) also decreased with depth; it was on the order of 10^−4^ between 0 and 20 cm depth in June, and between 0 and 30 cm in October (Fig 2B). Both parameters (*w* and *ε*) were higher in October due to the higher wind speed.

**Fig 3:**
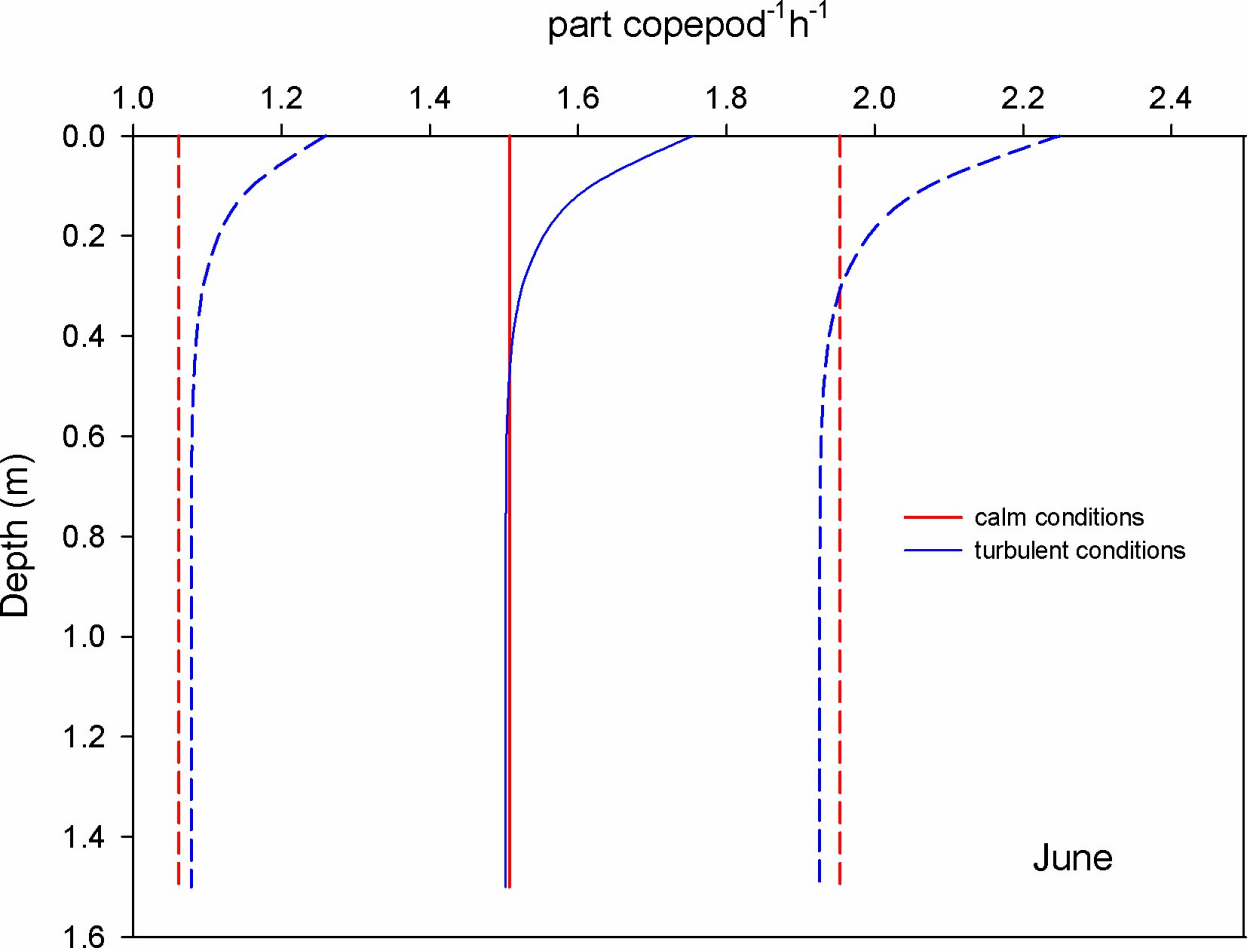
Mean encounter rate profiles (± SD in dotted lines) between copepods and microplastics (particles per copepod per hour) considering calm and turbulent conditions in the Sontecomapan lagoon during June.

**Fig 4:**
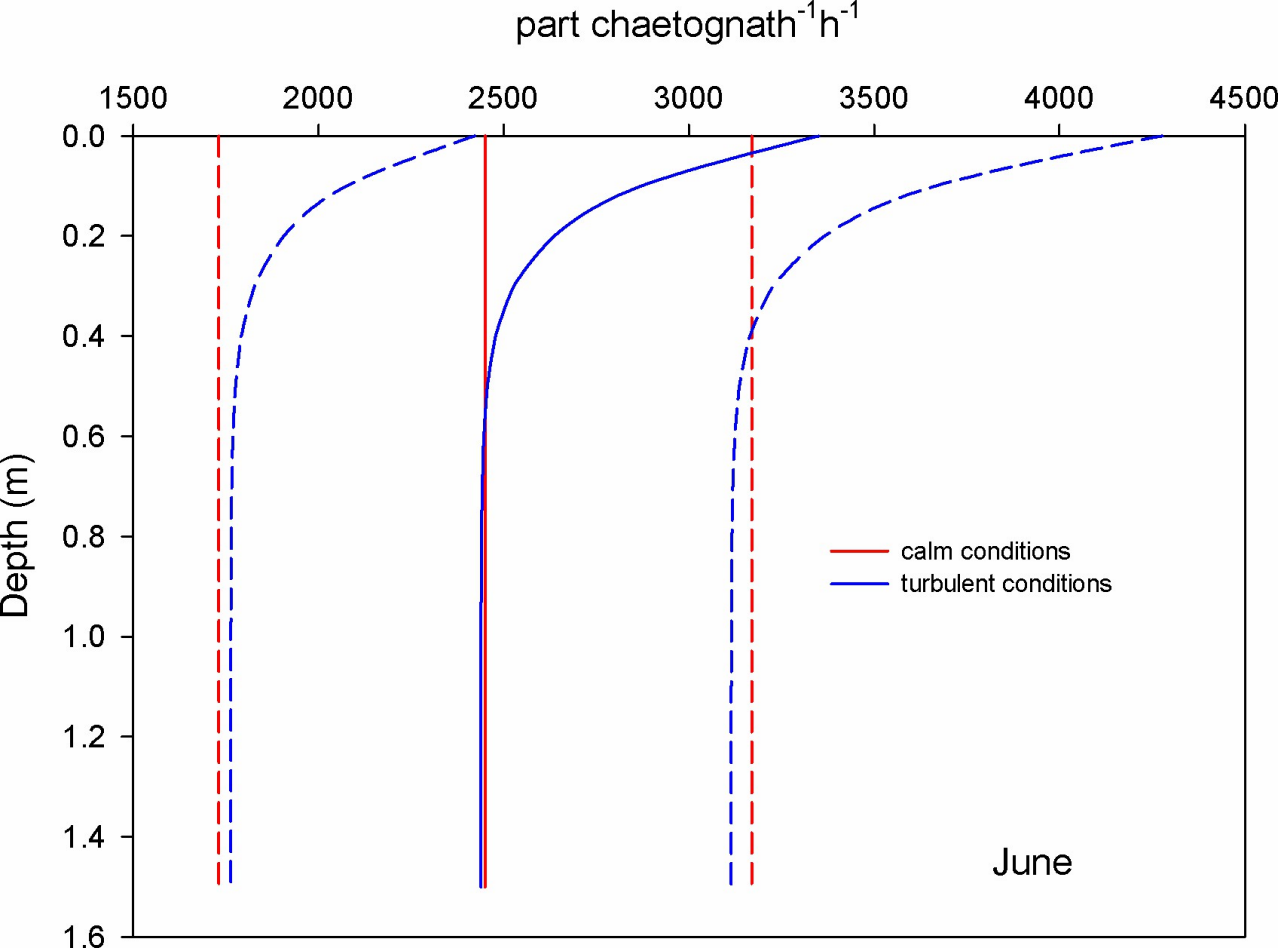
Mean encounter rate profiles (± SD in dotted lines) between chaetognaths and microplastics (particles per chaetognath per hour) considering calm and turbulent conditions in the Sontecomapan lagoon during June.

**Fig 5:**
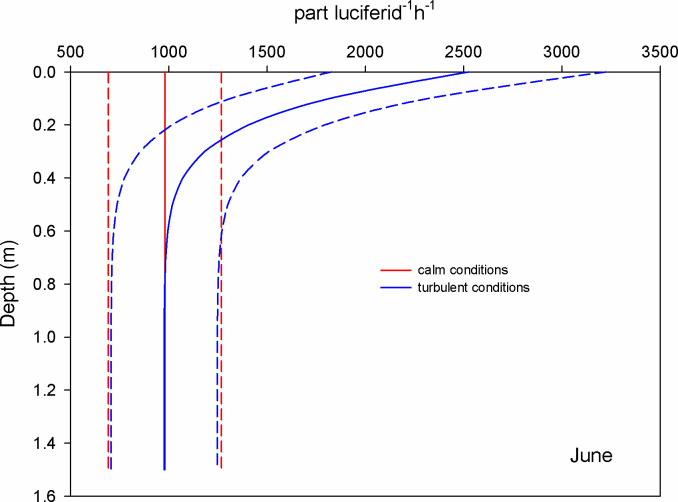
Mean encounter rate profiles (± SD in dotted lines) between luciferids and microplastics (particles per luciferid per hour) considering calm and turbulent conditions in the Sontecomapan lagoon during June.

During June, the estimation of encounters of copepods with microplastics revealed that at surface waters and during calm conditions (GS model), the mean encounter rate was 1.5 part copepod^-1^ h^-1^; under turbulent conditions (wind 0.8 m s^-1^), the encounter rate was 1.2 times higher (RO model) (Fig 3). For chaetognaths, the mean encounter rate was 2450 part chaetognath^-1^ h^-1^ considering calm conditions, but it increased 1.4 times when the wind blew (Fig 4). For luciferids, the surface encounter rate was 980 part luciferid^-1^ h^-1^ and was 2.6 times higher for turbulent conditions (Fig 5).

**Fig 6:**
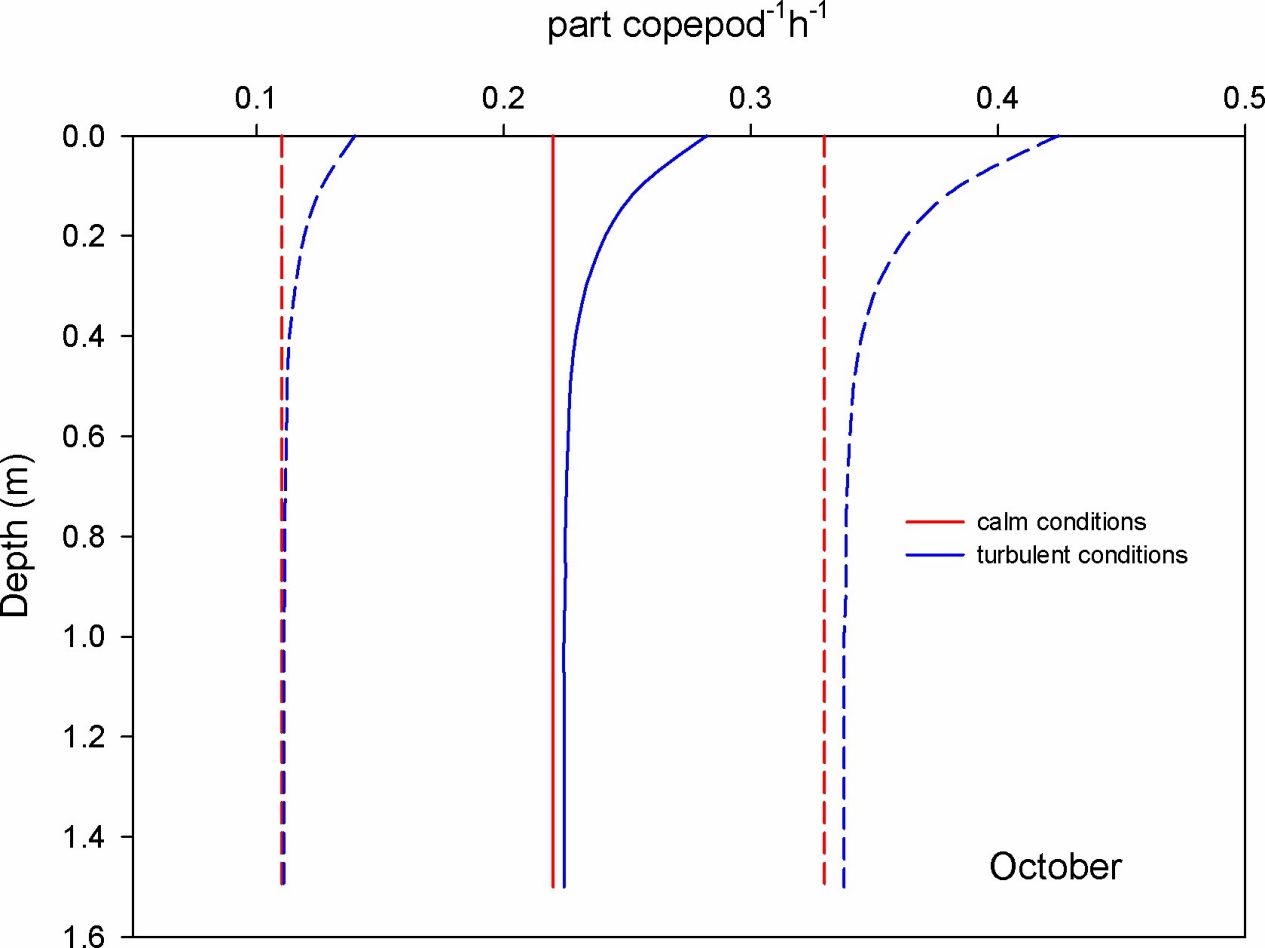
Mean encounter rate profiles (± SD in dotted lines) between copepods and microplastics (particles per copepod per hour) considering calm and turbulent conditions in the Sontecomapan lagoon during October.

**Fig 7:**
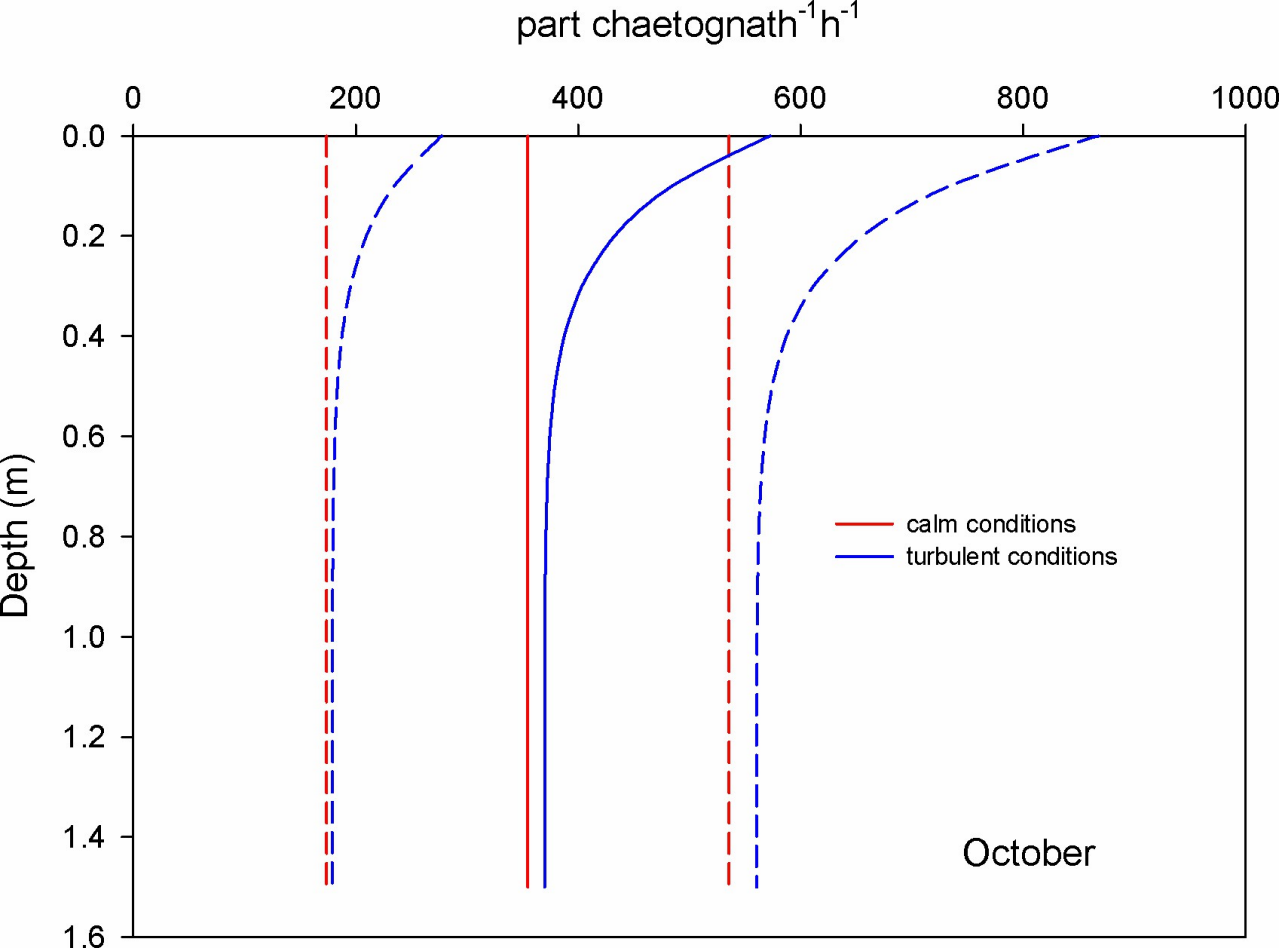
Mean encounter rate profiles (± SD in dotted lines) between chaetognaths and microplastics (particles per chaetognath per hour) considering calm and turbulent conditions in the Sontecomapan lagoon during October.

**Fig 8:**
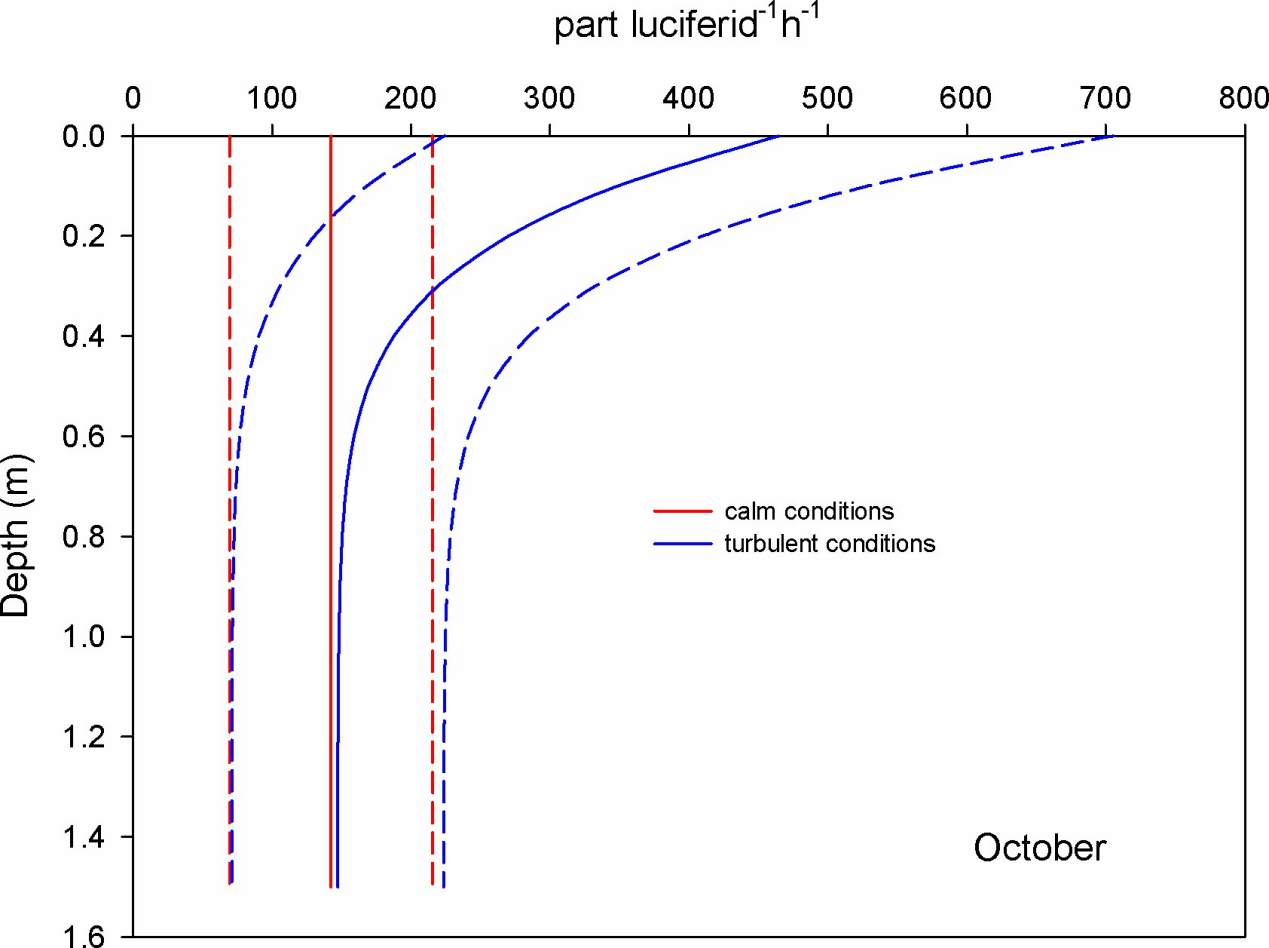
Mean encounter rate profiles (± SD in dotted lines) between luciferids and microplastics (particles per luciferid per hour) considering calm and turbulent conditions in the Sontecomapan lagoon during October.

In October, the mean encounter rate for copepods at surface waters was 0.2 part copepod^-1^ h^-1^ under the calm scenario (GS model); when the wind blows (1.12 m s^-1^) this value increased 1.3 times (RO model) (Fig 6). For chaetognaths, the mean encounter rate was 355 part chaetognath^-1^ h^-1^ for calm conditions and at the surface; this value was augmented 1.6 times under turbulent conditions (Fig 7). For luciferids, under calm conditions and at surface waters, the mean encounter rate value was 142 part luciferid^-1^ h^-1^; this value grows 3.3 times when the wind blows and causes turbulent conditions (Fig 8).

In all cases, mean rates using the RO model decreased with depth approaching the value of the GS model at 1.5 m depth, because turbulent velocity is almost null.

## Discussion

This study was based on the application of predator-prey ecological models to estimate the encounter rates between microplastics and zooplankton. In previous studies, an encounter rate had been defined as the ratio between microplastics in the seawater and zooplankton based on abundance [29, 44]; however, this relationship should be simply named microplastic: zooplankton ratio [45] to avoid confusion. Therefore, comparisons of this and previous studies in terms of encounter rates are not possible. As stated, for an organism to consume a particle, they must first find each other. Nevertheless, its consumption (or not) depends to a large extent on the feeding modes or the trophic position of organisms, as we show below.

The dominant copepod species in the lagoon, *Acartia tonsa*, can be viewed as an opportunistic species due to its high tolerance to abiotic conditions [46, 47]. In the Sontecomapan estuary and at surface waters, our estimations revealed that the encounters between microplastics (alleged prey) and copepods vary between 0.2 and 1.5 part copepod^-1^ h^-1^ (Figs 3 and 6) depending on wind conditions and microplastic concentration; these encounters might lead to the ingestion of the plastic particles, although the frequency of occurrence of this event is uncertain. Several laboratory and field studies have shown that copepods can consume microplastics [17, 29, 48]. However, for a feeding-current feeder copepod species, Xu et al. [49] found that the copepods rejected about 80% of the microplastics after encountering and touching them with their mouth parts. Regarding the feeding strategy, studies indicated that *A*. *tonsa* feed on small prey such as nauplii larvae, and flagellates, as well as phytoplankton [50, 51]. Depending on the availability of food, this species can switch between two capturing food methods: 1) filter suspension feeding strategy, where they generate micro-currents to encounter and capture non-motile prey by straining them on basket-shaped maxillae, 2) ambush feeding strategy, where they detect prey using hydromechanical signals [52, 53]. Blooms of phytoplankton favor the suspension feeding strategy; however, experimental studies showed that *A*. *tonsa* preferred motile prey [51, 52]. Microcosmos observations of *A*. *tonsa* and *Centropages typicus*, both having similar feeding modes, showed that moderate intensities of turbulence favor the selection of motile prey [52, 54]. Perhaps, micro-turbulence could cause confusion between microplastics and motile prey, resulting in accidental ingestion of the particles while feeding through the ambush feeding strategy; however, we cannot discard the ingestion of microplastics during the alternative feeding mode. Beyond this, encounters between microplastics and copepods may also result in the adhesion to the external surfaces of the animals, including the swimming appendages, antennae, furca, and feeding apparatus [17].

The high number of encounters between chaetognaths and microplastics (Figs 4 and 7) might result in either consumption or entanglement, with unknown rates. Field observations revealed the presence of microplastics in the gut of chaetognaths [30, 44, 55]; however, how these organisms ingested the microplastics is not understood. The only laboratory study trying to prove the ingestion of microplastics by chaetognaths revealed no evidence of direct consumption of plastic particles [17]. Studies on the feeding ecology of chaetognaths indicated that they feed on several moving zooplankton, consuming mainly copepods [56, 57]; they are ambush predators and can sense the micro-turbulence caused by their prey through tiny mechanoreceptor hairs [58]. An interesting ecological feature is that prey abundance does not influence their feeding rates because they probably reach a satiety state [56, 59]. In the field, most likely chaetognaths indirectly acquired the microplastics through the consumption of copepods or other small prey; however, they might accidentally consume the microplastics because of the possibility of confounding the hydrodynamic stimulus, especially under turbulent conditions. In accordance, Fuchs and Gerbi [60] stated that turbulence may cause a high interference in the motion signals between predators and prey. The question of how chaetognaths acquire microplastics in their habitat remains. More experimental studies concerning the consumption of microplastics by chaetognaths under different turbulence levels are needed to draw conclusions.

Owing to their swimming speed and perception distance, the encounter rates of luciferids showed an intermediate position between copepods and chaetognaths, but closer to the other secondary consumer, the chaetognaths. In the Sontecomapan lagoon, luciferids were all represented by *Belzebub faxoni* [37]. This species inhabits neritic and coastal zones and is especially abundant near the shoreline [61, 62]. These animals feed on zooplankton of moderate size and phytoplankton [62–64]; they are visual predators, but they can also perceive their prey through chemical or mechanical signals [62]. Fieldworks [44, 55] showed the presence of microplastics in the gut content of luciferids; however, it is unknown how luciferids acquired those contaminant particles. Based on the studies of Vega-Pérez et al. [62], we propose that most of the time, luciferids indirectly ingest microplastics by consuming their motile prey. Observations of Vega-Pérez et al. [62] exposed that adult luciferids prey more efficiently on metanauplii of *Artemia* than on newly hatched larvae because older larvae swim more actively and hydrodynamic disturbance can be detected by luciferids in a wider range. However, since turbulent conditions may confound the signals perceived by predators [60], the possibility of accidental ingestion of microplastics also exists.

For the three types of organisms here considered, the encounter rates with microplastics were higher in June than in October, despite the higher wind velocity in the last. Thus, the differences were mainly due to a greater microplastic concentration in June. Several studies revealed that the optimal level of turbulence for ingestion rates has a dome-shaped relationship [65–67], while other observations indicated negative effects of high turbulence on the ingestion rates [68]. Regarding predator-prey interactions in the plankton, the levels of turbulence (in units of the dissipation rate of energy, m^2^ s^-3^) at which the ingestion rates are higher, are on the order of 10^−7^ to 10^−5^ [66, 69, 70], similar to those estimated for the Sontecomapan lagoon in subsurface waters in both seasons (Fig 2B). Even if the encounters are higher, a relatively high speed between predators and prey also would imply more difficulty in capturing the prey; thus, an optimal level of capture must be a consequence of the balance of several forces: the level of turbulence, the ability of the predator to catch the prey, and the ability of prey to escape from predators [66]. Zooplankton must move into the water column to find the optimal level to catch their prey. Therein lies the question: does the direct consumption of microplastics by zooplankton occur at the same levels of turbulence as that for natural prey? Further experimental studies are needed to elucidate this problem. Ingested directly or indirectly, the consumption of microplastics by zooplankton may cause severe problems with their functional responses (i.e. the relationship between the feeding rate of a predator and the prey abundance). The shape of these curves depends on the prey density, the predator success rate, and the handling time per prey item. In turn, the success rate depends on the encounter rates, and the handling time refers to the sum of the time needed to capture and consume a prey item [25, 71]. In consequence, the time needed to process a plastic particle may be different from that wasted in a true prey, causing false satiety in the predator [72, 73] and thus, altering the population dynamics of both predators and prey.

Based on the encounter rate values estimated, one might expect that the organisms with higher values were the most affected by microplastics. Although the estimations presented here correspond to the characteristics of the Sontecomapan lagoon (wind conditions, concentration of microplastics), the attributes of the considered organisms do not change with the regions. In the few field studies that simultaneously explored the three types of organisms analyzed here, results indicated a higher concentration of the contaminant in luciferids and chaetognaths than in copepods [44, 55, 74]. In other marine areas considering only copepods and chaetognaths, contradictory results were found: higher concentration in chaetognaths [30, 31, 75, 76] or copepods [77, 78]. Based on these studies, we propose that secondary consumers (chaetognaths and luciferids) exhibit a major concentration of microplastics due to a double acquisition route: by consuming contaminated prey (microplastic ingested or adhered) or by direct consumption due to a mistake.

At this point, a question arises: what can happen if a predator and a plastic particle meet? We propose four scenarios. 1) Probably, in most cases, nothing will happen because microplastics must have certain characteristics to be bioavailable for the predator, the level of turbulence must be adequate, and the predator must still be hungry, among other ecological features. 2) After the encounter, microplastics can be rejected if they are unpalatable to predators [8, 49]. 3) Predators can accidentally ingest the microplastics as described above. 4) Microplastics could adhere to the bodies of zooplankton, especially in those with complex external morphology such as crustaceans, as indicated by Cole et al. [17]. The proportions in which zooplankton ingest or adhere microplastics to their bodies are unknown, but we think that there is a greater probability of adhesion than of ingestion, because adhesion does not depend on the feeding modes of zooplankton. Encounters between predators and prey, or alleged prey, do not always imply negative effects; however, encounters are necessarily the first step in the case of consumption or entanglement. Other than the danger of microplastics on the zooplankton, microplastics can be transferred through pelagic food chains [29, 48, 79] affecting organisms at higher trophic levels. In the Sontecompan estuary, the menace of microplastic pollution could be extended to anchovies, herrings, young mullets, or other planktivorous fishes of relevant ecological and commercial importance in the region. Given the rapid growth of anthropogenic activities and the continued input of plastics into water basins that potentially affect the health of organisms, it is important to reduce or avoid their use, and thus diminish their impact [80].

## Conclusions

This study models the number of encounters per unit of time between microplastics and three kinds of organisms having different feeding modes and trophic positions in the planktonic food webs: copepods, chaetognaths, and luciferids. The rate at which an individual zooplankter meets microplastics depends on the swimming speed and perception distance of the animal, as well as the concentration of microplastics in the water. Hence, organisms with higher encounters with microplastics were the chaetognaths, followed by the luciferids and the copepods. The small-scale turbulence enhances the encounters, especially in surface waters. Previous studies that simultaneously analyzed these three types of organisms found that the ingestion of microplastics per individual is higher in chaetognaths and luciferids than in copepods. Thus, we propose that secondary consumers (chaetognaths and luciferids) could be more affected because of the possibility of ingesting the microplastics directly due to confusion in the motion signals, or indirectly via contaminated prey. Once encountered, we think that four scenarios could be possible: no consequences (in most cases), rejection if microplastics are unpalatable to predators, ingestion of microplastics by accident, or external adhesion of microplastics to the body of organisms. Several questions arise on how zooplankton can accidentally ingest microplastics and the level of turbulence causing higher confusion, considering the different feeding modes of organisms.
